# Effects of Temperature, Growth Media, and Photoperiod on Growth and Toxin Production of *Azadinium spinosum*

**DOI:** 10.3390/md17090489

**Published:** 2019-08-22

**Authors:** Jane Kilcoyne, Amy McCoy, Stephen Burrell, Bernd Krock, Urban Tillmann

**Affiliations:** 1 Marine Institute, Rinville, Oranmore, H91 R673 Co. Galway, Ireland; 2 Biochemistry Department, School of Natural Sciences, National University of Ireland, H91 TK33 Galway, Ireland; 3 Alfred-Wegener-Institut für Polar- und Meeresforschung, Chemische Ökologie, Am Handelshafen 12, 27570 Bremerhaven, Germany

**Keywords:** azaspiracid, *Azadinium spinosum*, culturing, mass spectrometry, shellfish

## Abstract

Azaspiracids (AZAs) are microalgal toxins that can accumulate in shellfish and lead to human intoxications. To facilitate their study and subsequent biomonitoring, purification from microalgae rather than shellfish is preferable; however, challenges remain with respect to maximizing toxin yields. The impacts of temperature, growth media, and photoperiod on cell densities and toxin production in *Azadinium spinosum* were investigated. Final cell densities were similar at 10 and 18 °C, while toxin cell quotas were higher (~3.5-fold) at 10 °C. A comparison of culture media showed higher cell densities and AZA cell quotas (2.5–5-fold) in f10k compared to f/2 and L1 media. Photoperiod also showed differences, with lower cell densities in the 8:16 L:D treatment, while toxin cell quotas were similar for 12:12 and 8:16 L:D treatments but slightly lower for the 16:8 L:D treatment. AZA1, -2, and -33 were detected during the exponential phase, while some known and new AZAs were only detected once the stationary phase was reached. These compounds were additionally detected in field water samples during an AZA event.

## 1. Introduction

**Figure 1:**
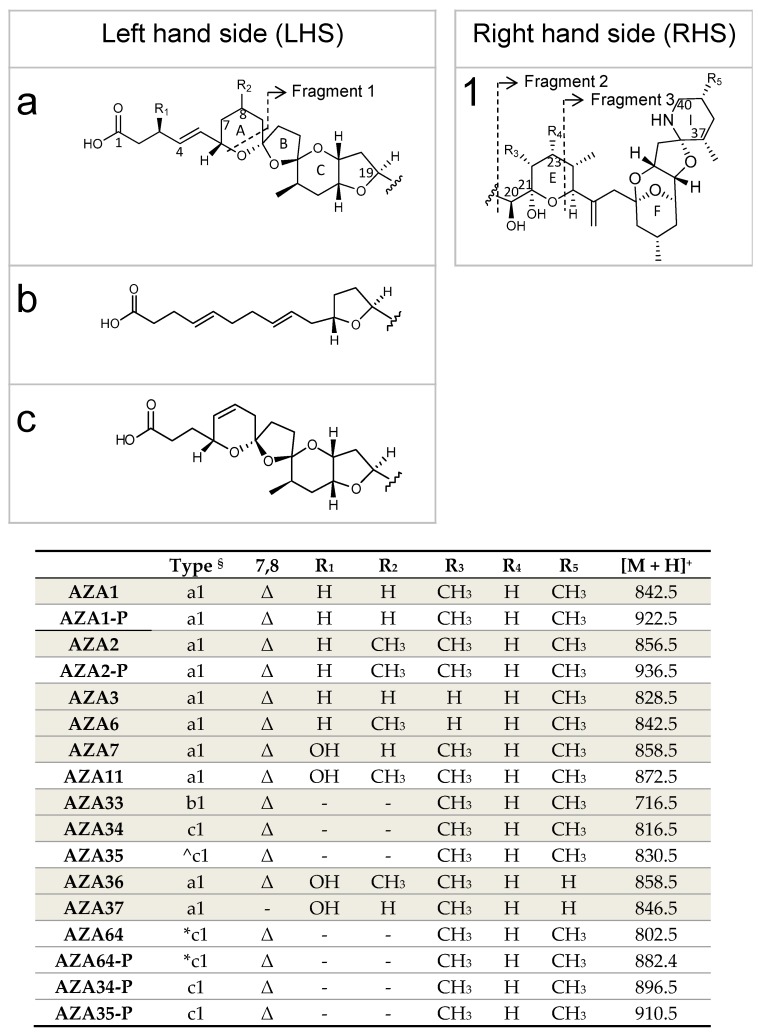
Structures and protonated masses of AZAs. Compounds highlighted in grey have had their structures confirmed by NMR. ^§^ The type refers to variations of the LHS and RHS parts of the molecule. ^ Putatively has an additional CH_2_ at C6 of AZA34. * Putatively missing a CH_2_ between C1–C4. Δ refers to the presence of a double bond between C7–C8.

Since the first identification and characterisation of azaspiracid-1 (AZA1) in 1998 [1], following the consumption of contaminated shellfish that led to a poisoning incident in 1995, more than 60 additional analogues have been reported [2,3,4,5,6,7]. Only AZA1, -2, and -3 are regulated (160 µg kg^−1^ AZA1 equivalents in raw whole flesh) [8]. The AZAs have a global distribution, but to date they have been most problematic for the Irish shellfish industry with blooms of *A. spinosum* occurring annually on the west coast of Ireland, resulting in frequent shellfish farm closures [9]. So far, 26 AZAs have been identified in *A. spinosum*, *A. poporum*, *A. dexteroporum*, and *Amphidoma languida* strains [7]. Of these, only six have been purified and fully characterised [10,11], Figure 1. Many of the other AZAs identified are shellfish metabolites [5], while some have been reported as artefacts [12,13].

The number of species within the Amphidomataceae (Dinophyceae) family is fast expanding. By far not all species produce AZAs, but for the currently known four species that do, the profiles are varied. Various strains of *A. spinosum* produce the toxins AZA1, -2, -33, -34, and -35 [10], but other strains representing a new ribotype of *A. spinosum* collected from the Norwegian coast, have a completely different toxin profile consisting of AZA11, -50, -51, and their phosphorylated conjugates [14].

AZAs are cytotoxic against several mammalian cell lines [15] and induce severe gastrointestinal illness in humans [16]. The primary mode of action has remained elusive, but they have been found to be potent activators of c-Jun-*N*-terminal kinase (JNK) and caspases [17,18], an effective modulator of intracellular cAMP and calcium levels [19], potassium channel blockers [20], and they induce cell lysis via apoptotic pathways [21]. Several toxicological and biological studies have highlighted the potential of dinoflagellate-derived biotoxins as promising pharmacological effectors and/or biological investigation tools. As such, further pharmacological/toxicological investigations may lead to therapeutic/biotechnological applications for AZAs [22]. 

The availability of purified toxin is critical to produce reference materials that are required to ensure effective monitoring of these toxins in shellfish, plankton, and water samples. Pure toxins are further required for developing new analytical methods and for use in other areas of research, e.g., toxicology, pharmacology, and the development of biosensors. Isolation of AZAs from shellfish [23,24], a marine sponge [25], and cultures of *A. spinosum* [10] and *A. poporum* [11] have been reported. Purified AZAs (Figure 1) for the production of certified reference materials (CRMs) [26], and for toxicological studies [16,27], have been sourced from both contaminated shellfish and algal biomass, with the majority coming from contaminated shellfish, sourced after a major AZA incident in the northwest of Ireland in 2005. Currently, the availability of purified AZAs is limited to µg–mg amounts, thereby restricting further biochemical investigation, particularly mode of action and pharmacological studies. AZAs have been successfully synthesised, but chemical synthesis of these compounds is complex [28,29,30]. Apart from chemical synthesis, some AZAs such as AZA3 and -6 can only be sourced from contaminated shellfish, being shellfish metabolites of AZA1 and -2, respectively [31], and therefore purification from shellfish is still required.

As algal biomass is less complex than shellfish, purification requires fewer steps and is therefore the preferred option. Optimisation of culture conditions is critical for enhanced efficiencies. Reports of *A. spinosum* AZA cell quotas in batch culture ranged from 0.1–220 fg cell^−1^ [14,32,33,34]. Growing a strain of *A. spinosum* (3D9) [35], isolated from the east coast of Scotland, at a lower temperature was found to enhance toxin cell quotas (20-fold) compared with higher temperatures. Additionally, toxin cell quotas were enhanced when cultures were aerated [34]. The impact of growth media was also assessed, however, no significant differences were observed between the tested media [34]. A similar study was performed on strains of *A. poporum* [36], isolated from the south China Sea, where AZA2 was the predominant toxin. In that study, the impacts of nitrate and phosphate concentrations, different sources of nitrogen and media were investigated with higher cell quotas obtained in the phosphate limited cultures. Culturing of the 3D9 strain of *A. spinosum* at a larger scale was also performed in two 100 L photobioreactors [37]. From 1200 L biomass yields of ~13 mg of AZA1 and ~3 mg AZA2 were attained [37]. From the harvested algal biomass, AZA1, -2, -33, and -34 were purified and fully characterised [10]. 

The aim of this study was to further improve culturing conditions for increasing toxin yields in *A. spinosum.* We assessed the impacts of temperature, different culture media (different nutrient compositions and concentrations), and photoperiod on cell densities and toxin cell quotas. We further describe changes in toxin profiles over the different growth phases and identify some new AZAs. This work has offered insights into what factors influence toxin cell quotas, and to date no data have been reported on how toxin profiles can change after the stationary phase has been reached.

## 2. Results and Discussion

### 2.1. Impact of Temperature 

**Figure 2:**
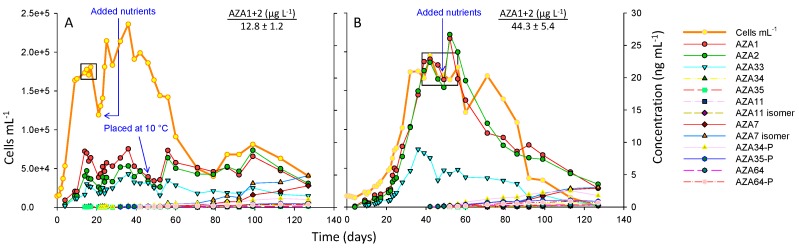
*A. spinosum* growth curves at (**A**) 18 °C and (**B**) 10 °C in the 5 L culture flasks. Boxes indicate data points used to calculate fg cell^−1^ (Tables S1 and S2) and AZA1+2 µg L^−1^ values.

In the 18 °C treatment cell densities increased rapidly over 11 days with a growth rate of 0.22 d^−1^ (day 2–11), reaching ~180,000 cells mL^−1^ at the start of the stationary phase and after ~17 days began to decline (Figure 2A). Peak of toxin cell quota (AZA1) was ~44 fg cell^−1^ (Table S1). After 22 days, nutrients were added to the culture resulting in resumed cell growth. After 49 days, the culture flask was placed in the 10 °C incubator to determine if toxin production would be enhanced at the lower temperature. The results indicate that toxin cell quotas increased after this change in temperature, with an increase in toxin levels occurring after ~56 days, suggesting that a low temperature stress can enhance toxin cell quotas. After 80 days, there was an apparent increase in cell densities that continued for ~20 days, which was then followed by a decline. Generally, it has to be kept in mind that the addition of nutrients and the change in temperature was performed on one replicate and without a proper control, thus all respective results are only indicative. After 130 days the culture was harvested leading to a more comprehensive overview of toxin profiles that is presented in Section 2.4.

In the 10 °C treatment, the growth rate was slower at 0.12 d^−1^ (day 21–32), taking 32 days to reach the stationary phase at 174,000 cells mL^−1^ (Figure 2B), which continued for ~30 days (whereby cell counts remained constant), followed by a phase of decreasing cell densities (decline phase). After 49 days nutrients were added to the culture with no significant increases in cell growth. Peak toxin cell quota (AZA1) was ~135 fg cell^−1^ (Table S2). After 127 days the culture was harvested. 

The results for the 10 °C culture flask show that toxin cell quotas remain steady over the exponential growth phase, up to 22 days, and then increase slightly in the later exponential phase (from day 23 to 28). In both the 18 and 10 °C culture flasks toxin cell quotas increased from the exponential phase to the early stationary phase (~2-fold), and again in the late stationary phase (~2-fold), at which point maximum yields are achieved (Figure 2; Tables S1 and S2). 

Small/medium scale studies assessing environmental factors affecting growth, maximum cell densities, and AZA cell quotas of *A. spinosum* were previously performed [34]. In these studies highest cell quotas were achieved at 10 °C, and with aeration. The higher cell quotas at the lower temperatures (10 and 15 °C) were due to induced higher biosynthesis of AZAs, but also due to slower growth and higher toxin accumulation [34]. In this study toxin cell quotas were higher by ~3.5-fold at 10 °C than at 18 °C, while in the Jauffrais et al. [34] study, a 16-fold difference in cell quotas was observed (220 fg cell^−1^ vs. 14 fg cell^−1^ at 10 and 18 °C, respectively). Another notable difference is that in this study there was little difference in the maximum cell densities reached at the two temperatures (174,000 vs. 180,000 cells mL^−1^ at 10 and 18 °C, respectively), whereas in the Jauffrais et al. [34] study, maximum cell densities had been ~50,000 and 90,000 cells mL^−1^ at 10 and 18 °C, respectively. In both these studies, toxin yields were higher at 10 °C (~44 vs. 11 µg L^−1^ [34] AZA1+2) compared to 18 °C (~13 vs. 1.3 µg L^−1^ [34] AZA1+2). In larger scale culturing studies by Jauffrais et al. [37] (using two stirred 100 L photobioreactors in series) ~13 µg L^−1^ AZA1 + 2 had been achieved when *A. spinosum* was grown at a temperature of 18 °C. Cell densities reached ~200,000 cells mL^−1^, a significant increase from the smaller batch culture studies [34], and similar to the cell densities (and yields) achieved in this study. 

Although growth at low temperature consistently increased AZA yield, it has to be kept in mind that growing *A. spinosum* at lower temperatures on a large scale could be costly, requiring a photobioreactor equipped with a chiller or larger temperature-controlled walk-in incubators. The increase in toxin production when *A. spinosum* was transferred from 18 to 10 °C, observed in this study (Figure 2A), suggests future studies could focus on assessing whether a low temperature stress, during the late exponential or stationary phase, could produce similar increases in toxin yields, thereby reducing time and operation costs. 

### 2.2. Impact of Growth Media 

**Figure 3:**
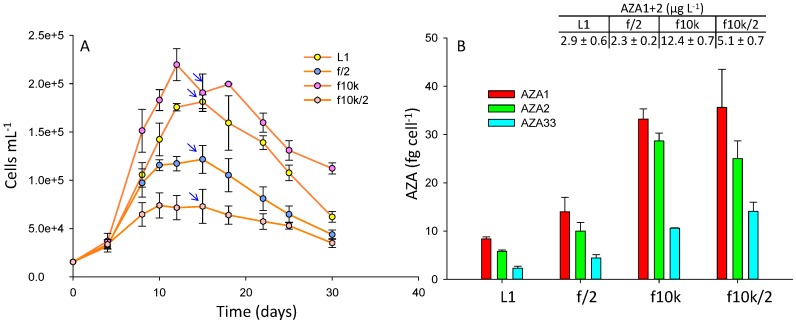
*A. spinosum* growth curves (**A**) and AZA cell quotas (**B**) in L1, f/2, f10k, and a diluted (x2) f10k media. Arrows indicate point at which AZA cell quotas and yields (AZA1+2 µg L^−1^) were calculated.

Four culture media were tested: L1, f/2, f10k, and a diluted (2-fold) f10k. The f10k medium has a 4.5-fold lower amount of most ingredients compared to L1 and f/2 (Table S3). Cell densities were 1.2- and 1.8-fold higher in the f10k (reaching a maximum of ~220,000 cells mL^−1^) compared with L1 (~180,000 cells mL^−1^) and f/2 (~120,000 cells mL^−1^), respectively (Figure 3A), while the diluted f10k gave the lowest cell densities (reaching a maximum of ~66,000 cells mL^−1^). There was no significant difference in AZA cell quotas between the f10k and the diluted f10k (*p* = 0.26), while significant differences (*p* < 0.05) were observed between the f10k, L1, and f/2 media, with the f10k and diluted f10k giving 4–5- and 2.5–3-fold higher cell quotas compared with L1 and f/2, respectively (Figure 3B). The highest AZA1+2 yields (µg L^−1^) were obtained in the f10k medium (Figure 3). To ensure these differences were not due to media matrix interferences in the LC-MS, which can be problematic in the analysis of these compounds [38], matrix matched standards were prepared and analysed, and the results showed that there was no significant difference in the responses (*p* = 0.09) between the media (Figure S1). 

L1 medium is derived from f/2 but has additional trace elements [nickel sulfate (NiSO_4_.6H_2_O), sodium orthovanadate (Na_3_VO_4_), and potassium chromate (K_2_CrO_4_), Table S3]. The f10k medium has lower amounts of all the nutrients with the exception of selenium (H_2_SeO_3_). Selenium is common to both the L1 and f10k but is not present in f/2. Given that cell densities were significantly higher in both the f10k and L1 media relative to f/2 suggests selenium is an important micro-nutrient for enhancing *A. spinosum* final cell yield. In the studies performed by Jauffrais et al. [34] cell densities increased from ~90,000 cells mL^−1^ in small scale batch culture to ~200,000 cells mL^−1^ in larger scale photobioreactors [37] using the same medium, but in the larger scale study the medium had been enriched with selenium. In a previous study, looking at yessotoxin production in *P. reticulatum*, the addition of selenium increased growth rate, cell yield and toxin production significantly [39]. AZA cell quota was lowest in the L1 medium (Figure 3B), which might indicate the presence of L1 trace elements, missing in either f/2 or f10k (Table S3), may play a role in diminishing toxin synthesis.

A potential role of nutrient limitation is more difficult to evaluate. In a previous study using continuous cultures, a 2-fold dilution of K-medium led to an expected decrease of steady state cell density, but also to a ~1.2-fold increase of toxin cell quota in *A. spinosum* [34]. In the present batch culture study, final cell yield was also negatively impacted when the f10k was diluted 2-fold, but there was no significant difference in toxin cell quotas when compared to the full strength f10k medium (Figure 3B). In the Li et al. [36] study AZA2 cell quotas in *A. poporum* reduced significantly when concentrations of phosphate were increased, while increases in nitrogen had no impact.

In the Jauffrais et al. [34] study there had been no significant difference between the investigated media (K medium [40] with and without tris buffer, L1, and a L1 medium with a soil extract) although the authors did report a higher growth rate in the K medium compared with L1, and a slightly higher AZA cell quota in the K medium that contained the tris buffer. Lower cell densities had been obtained in the L1 medium (~100,000 cells mL^−1^) [34] than in this study (~180,000 cells mL^−1^). Another study on two strains of *A. poporum* [36] assessed the impacts of nitrate and phosphate concentrations, different sources of nitrogen, and media (L1-Si, f/2-Si, and K-Si). No significant differences in growth and toxin cell quotas were observed between the media for one strain, but the second strain produced higher cell densities in the L1-Si media. Cell quotas had also been higher in the L1-Si media compared to the f/2-Si media for one of the strains (~2-fold), while there had been no significant difference in the second strain. In this study, higher cell quotas (~1.5-fold) were obtained in f/2 relative to L1, although AZA1+2 yield was higher in the L1 medium (Figure 3). 

Toxin cell quotas were ~5-fold higher in the 5 L culture flasks (Figure 2, ~44 fg cell^−1^ AZA1), compared with the 250 mL culture flasks (Figure 3, ~8 fg cell^−1^ AZA1), under the same conditions (L1, 18 °C, 12:12 L:D, and a light intensity of 32 µmol m^−2^ s^−1^). It is unclear as to why these differences were observed, but given that these experiments were performed at different times of the year, a variable to consider is the source of seawater used to grow the microalgae. The nutrients profile of seawater can differ significantly between location and time of year. More important, however, is to consider that virtually nothing is known with respect to temporal changes/variability in growth and/or toxin production potential of Amphidomatacean cultures, e.g., to potential rhythmic/seasonal cycles or long-term changes in response to the artificial laboratory environment, where competitive or more complex food web interactions are removed. We observed that toxin production in one strain of *A. spinosum* ceased after several years in culture (unpublished information). High variability (differences of up to ~20-fold) in AZA2 cell quota is obvious in the data presented by Li et al. [36], for different sets of experiments (under the same conditions) using *A. poporum*. These variations, both within and between studies, highlight the challenges that remain with respect to controlling conditions for toxin production.

### 2.3. Impact of Photoperiod

**Figure 4:**
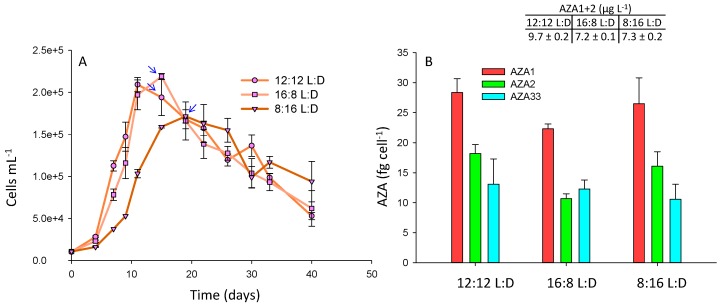
*A. spinosum* growth curves (**A**) and AZA1, -2, and -33 cell quotas (**B**) under photoperiods of 12:12, 16:8, and 8:16 L:D. Arrows indicate point at which AZA cell quotas and yields (AZA1+2 µg L^−1^) were calculated.

In this study, photoperiods of 12:12, 16:8, and 8:16 L:D were assessed. The cultures were grown in f10k, after the results in the growth media experiments (Section 2.2) indicated that higher AZA cell quotas were achieved in this medium. The growth curves were very similar between the three treatments, with growth rates (4–11 days) of 0.28, 0.30, and 0.26 d^−1^ for 12:12, 16:8, and 8:16 L:D, respectively (Figure 4A), but toxin cell quotas were slightly higher (*p* < 0.05) for AZA1 (1.3-fold), -2 (1.7-fold), and -33 (1.1-fold) at the 12:12 photoperiod relative to the 16:8 L:D treatment (Figure 4B). There was no significant difference (*p* = 0.55) in toxin cell quotas between the 8:16 L:D and 12:12 photoperiods (Figure 4B). A difference (*p* < 0.05) in the AZA1:AZA2:AZA33 ratio was observed at the 16:8 L:D cycle, with a slightly lower level of AZA2 and a higher level of AZA33 relative to AZA1 (Figure 4B). There was no significant difference in toxin cell quotas between the 12:12 and 8:16 L:D treatments, but cell densities were higher under the 12:12 L:D treatment, and therefore yields were greater (Figure 4).

In the Jauffrais et al. [34] study light intensity had been studied (with no differences in cell growth or toxin cell quotas observed when comparing irradiances ranging between 50–250 µmol m^−2^ s^−1^), but photoperiod had not been assessed. Larger scale culturing of *A. spinosum* in two 100 L photobioreactors had used a photoperiod of 16:8 L:D [37]. The results in this study indicate that this light cycle may have slightly limited toxin production.

### 2.4. Toxin Profiles 

The 3D9 strain of *A. spinosum* is known to produce AZA1, -2, -33, -34, and -35 [10]. In all experiments AZA1, -2, and -33 were detected in the exponential phase of growth. AZA34 and -35 were only detected once the stationary phase was reached (Figure 2). 

The AZA1/AZA2 ratio in the temperature and growth media trials ranged from ~1 to 1.5, while the AZA1/AZA33 ratio was ~3 (Figure 2 and Figure 3). The AZA1/AZA2 ratio reported in the Jauffrais et al. [34] study had been significantly higher (~3–4-fold). These results also demonstrate how toxin production can vary significantly between experiments. 

Figure 2 shows a decline in toxin amounts that corresponded to the decline in cell numbers that was particularly noticeable for the 10 °C culture flask (Figure 2B). It was suspected that this could be due to adsorption of toxins onto the stack walls (polystyrene) after release into the media following cell rupture. The results in Table 1 suggest that this was the case with up to ~65% of the toxins being extracted (with MeOH) from the 10 °C culture flask, while adsorption was lower at 18 °C. Adsorption was highest for AZA33 (that corresponds with a higher lipophilicity) and was significantly higher (~2-fold) at 10 °C compared to 18 °C (Table 1). These results show that adsorption of toxins onto culture flasks should be considered when performing such experiments.

**Figure 5:**
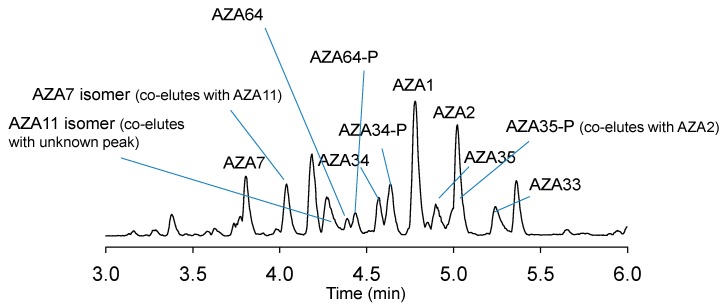
Total ion chromatogram (*m/z* 200–1200) of *A. spinosum* HP20 resin extract showing detected AZAs.

**Figure 6:**
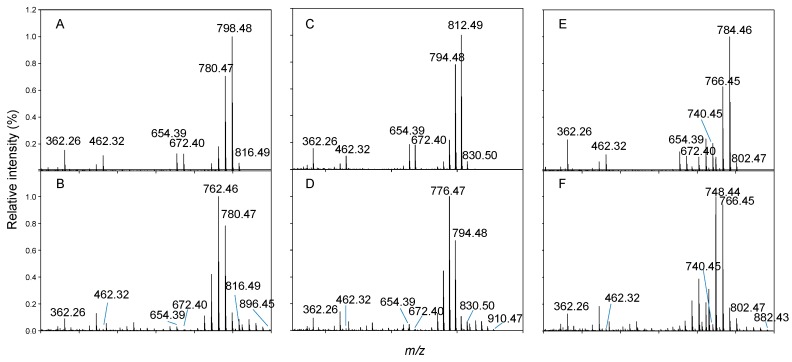
Mass spectra of (**A**) AZA34, (**B**) AZA34-P, (**C**) AZA35, (**D**) AZA35-P, (**E**) AZA64, and (**F**) AZA64-P at a collision energy of 50 V.

A new AZA analogue with a *m/z* of 802.4742 (Figure 5 and Figure 6; Table 2), named AZA64, was detected in the HP20 resin extracts from the 5 L flasks that comprised ~10% that of AZA1. This value of *m*/*z* was consistent with C_44_H_67_NO_12_^+^ (Δ 0.74 ppm). Its [M + H]^+^ was 14 mass units lower indicating a difference of a CH_2_, likely missing in the carboxylic side chain (Figure 1). The MS/MS spectrum (Figure 6) displays four water losses from the molecular ion peak, similar to AZA34 [10], so should have a similar number of hydroxyl groups. The RDA cleavage (*m/z* 672), 462, and 362 fragment peaks are also similar to AZA34 (and AZA1), and therefore should be the same structurally up to the A ring. The spectrum also shows a CO_2_ loss (-44), which would normally indicate a C3 hydroxylation. The close proximity of the carboxyl group to the oxygen in the A ring could explain the loss of the CO_2_. A tentative structure for this compound is proposed (Figure 1), however, NMR data will be required to confirm. 

AZA34 and -35 had been previously identified in the harvested biomass of a bulk culture of *A. spinosum* [10,37]. The structure of AZA34 had been confirmed by LC-MS/MS and NMR, while AZA35 had been proposed as being similar to AZA34, but with a methyl group at C6 of AZA34 (equivalent to C8 in AZA1) [10], Figure 1. Levels of these analogues had been higher in the permeate (filtrate) than in the retentate, and it was suggested that these analogues were produced by the cells and then excreted [37]. It has been suggested that AZAs are generated by cyclisation of a long carbon chain assembled, primarily from acetate units via polyketide synthase enzyme clusters. The structures of AZA33 and -34 further suggested that assembly is from the amino end of the molecule given that the C19–C40 substructure (Figure 1), including the relative configuration and all substituents, were identical to AZA1 and -2 [10]. 

Three other compounds, which are suspected to be conjugates of AZA34, -35, and -64, were detected after reaching the stationary phase (Figure 5, Table 2 and Table 3). Each spectrum was similar to the nonconjugated form but differed by having a mass difference of 79.96 Da (Figure 6 and Table 2), indicative of a phosphorylation. The RDA cleavage and *m/z* 462 fragment peaks were significantly less prominent compared with the non-conjugated analogues (Figure 6). There were several water losses from the [M + H]^+^ peak, as well as the loss of a CO_2_. The phosphorylated conjugate peaks (which were fronting) eluted just slightly after the nonconjugated form (Figure 5 and Figure S2–S4). Levels were additionally higher (1.6–5.4-fold) than the nonconjugated analogues (Table 3), however, these values are only indicative given that they were quantitated against an AZA1 standard and may have different response factors in LC-MS/MS analysis. 

To determine whether the phosphorylation was occurring at the C20–C21 cis diol region of the AZAs (Figure 1), a cleavage with periodate was performed. Periodate cleaves the diol moiety of AZA at C20–C21, giving the structurally diagnostic C20–C21-diol-cleavage product (*m/z* 448). Treatment of the *A. spinosum* HP20 resin extract (from the 5 L culture flasks) with periodate resulted in the disappearance of AZA34, -35, and -64 (and the observation of the *m/z* 448 cleavage product), but no changes were observed for the phosphorylated conjugates (Figures S2–S4). These results strongly suggest that phosphorylation is occurring at the C20–C21 cis diol region of the AZAs. 

It is unclear why AZA34 and -35 are only detected once the stationary phase is reached. It may be that they were not detected during the exponential phase due to levels being below the LC-MS method limit of detection (~0.3 ng mL^−1^). Regardless, levels increase once the stationary phase is reached, particularly for the phosphorylated conjugates (Figure 2 and Figure S5). As these are truncated analogues of AZA1 and -2, respectively, they may be produced by the cells after a metabolic shift in biosynthesis once the stationary phase is reached. This may also account for the production of AZA64. AZA33 is also a truncated AZA analogue, but as it is produced during the exponential phase suggests it is produced as part of the same biosynthetic pathway as AZA1 and -2.

Generally, the formation of AZA phosphates is poorly understood. In the *A. poporum* cultures, from the southeast Pacific, both AZA11 and the phosphate form were present and the authors concluded that the phosphorylation was occurring de novo within the cells [41]. Moreover, Argentinian strains of *A. poporum* had been found to produce AZA2 and its phosphorylated conjugate (~3.5% that of AZA2) in culture [42], and phosphorylated conjugates of AZA1, -2, -11, -33, -50, and -51 had been detected in Norwegian *A. spinosum* strains [14]. The MS/MS spectra of the phosphorylated AZAs showed similar CID fragments as the nonconjugated forms, with the RDA cleavage (fragment 1) and 462/448 peaks (fragment 2) being less intense in the phosphorylated AZA spectra. Similarly, in this study, the RDA cleavage (*m/z* 672 and 654) and 462 peaks were less intense in the phosphorylated spectra (Figure 6). It is unclear as to why phosphorylated conjugates were only detected for AZA34, -35, and -64 in the cultures and not for the other AZAs (AZA1, -2, -33, -7, and -11), Figure 2 and Figure S5.

In the harvested samples (from the 5 L culture flasks) other AZA-type peaks were visible that were shown by LC-MS/MS to be AZA7, an isomer of AZA7, AZA11, and an isomer of AZA11 (Figure 5, Figures S6 and S7; Table 3). AZA7 and -11 were initially identified in shellfish as C3 hydroxylated metabolites of AZA1 and -2, respectively [2]. Similar to AZA34, -35, -64, and their phosphorylated conjugates, these compounds were only detected after reaching the stationary phase (Figure 2 and Figure S8). AZA11 has been reported in strains of *A. poporum* isolated from the south China Sea, the southeast Pacific [3], and the Mediterranean [43], and *A. spinosum* ribotype B strains collected off the Norwegian coast [14]. The detection of AZA7, an AZA7 isomer, and AZA11 isomer, is the first reporting of these AZA analogues in cultures of *A. spinosum*. For both AZA7 and -11, the isomers eluted ~0.2 min later (Figures S6 and S7). The spectra of AZA7 and its isomer were identical apart from a lower *m/z* 796.5 (loss of CO_2_) peak intensity in the isomer spectrum (Figure S6), suggesting isomerisation is occurring at the carboxyl end of the molecule, possibly at C3. Similarly, the spectra of AZA11 and its isomer were identical apart from a lower 810.5 (loss of CO_2_) peak intensity in the isomer spectrum (Figure S7). In this study, all of these C3 hydroxylated analogues were detected only after the stationary phase was reached, suggesting (for the *A. spinosum* strain 3D9) they could be degradation products, and are not necessarily being produced by the cells. Jauffrais et al. [34] reported an overall decrease in AZAs (by 31%) from day 14 to 17 of culture growth – this decrease may be due to the formation of these C3 hydroxylated AZAs. Levels of the compounds detected once the stationary phase was reached (AZA7, -11 and their isomers, and AZA34, -35, -64 and their phosphorylated conjugates) increased over the testing period (Figure 2, Figures S5 and S8). 

In order to determine if these toxin profiles were present in field water samples, SPATT (solid phase adsorption toxin tracking) extracts, generated in previous studies carried out by Fux et al. [44,45], were reanalysed by LC-MS/MS, also targeting the novel AZA variants. All the AZAs detected in this study were found in the SPATT extracts (Table 4). The proportions of the AZAs detected only after the stationary phase was reached, relative to AZA1 and -2, were lower in the SPATTs than in the cultures, apart from AZA64 which was ~10% that of AZA1 in both the culture and SPATT extracts (Table 3 and Table 4; Table S4). Additionally, phosphorylated conjugates of AZA1 and -2 (~6% that of the parent analogues) were detected in the SPATT extracts (Figures S9 and S10), but not in the cultures (Table 3 and Table 4). Phosphorylated conjugates of AZA7, -11, and -33 were not detected either in the culture or SPATT extracts.

## 3. Materials and Methods

### 3.1. Culture Conditions

The stock culture of *A. spinosum* (3D9 strain) [35] was grown in L1 media at 18 °C. Cultures were grown in 5 L (Corning^®^ cellSTACKs^®^, Lowel, MA, containing 2 L culture) and 250 mL (Sarstedt, Nümbrecht, Germany, containing 100 mL culture) culture flasks. 

#### 3.1.1. Culture Sampling 

For cell densities (cells mL^−1^), the cultures were sampled by transferring 500 µL of well mixed culture into a 1.5 mL centrifugation tube containing 400 µL seawater and 100 µL lugols iodine. As cultures became denser the ratio of seawater to culture increased. Cells were counted on a Sedgwick rafter by visual microscopy (Olympus model BX53, Madison, WI, USA).

For LC-MS/MS analysis of AZAs, the cultures were sampled by transferring 100 µL of well mixed culture into an insert vial containing 50 µL MeOH, vortex mixed for 0.5 min, and stored at −18 °C until analysis. 

Growth rate µ (d^-1^), as the exponent of the exponential equation, was calculated from cell density time series data by exponential regression of cell counts versus time, for a defined period of exponential growth using Microsoft Excel.

#### 3.1.2. Impact of Temperature

The stock culture was inoculated (200 mL stock culture in the exponential phase) into 1800 mL L1 media contained in 5 L culture flasks and placed in incubators with a photoperiod of 12:12 light:dark (L:D), a light intensity of 32 µmol m^−2^ s^−1^, and at two temperatures—10 and 18 °C. After 22 and 46 days of growth nutrients (1.5 mL of stock phosphate solution (5 g L^−1^), 1.5 mL of nitrate stock solution (75 g L^−1^), 1.5 mL L1 trace metals solution [46], and 0.75 mL f/2 vitamins solution [47]) were added to the 18 °C and 10 °C treatments, respectively. After 49 days of growth the culture grown at 18 °C was placed in the 10 °C incubator. 

#### 3.1.3. 5 L Culture Flask Harvesting

The 5 L culture flasks (Corning^®^ cellSTACKs^®^) were harvested by filtering through 3 µm polycarbonate (TSTP, Merck Millipore, MA, USA) filter paper. A HP20 resin (5 g) SPATT [48] was added to the filtrate and left stirring for 3 days (when analysis of the filtrate indicated that >95% of toxin had been adsorbed onto the resin). The filter papers were extracted by adding 9 mL MeOH and vortex mixing for 1 min. This was repeated 4 times, with the extracts being transferred into a volumetric flask and made up to 50 mL with MeOH. The culture flask was rinsed twice with 100 mL MeOH combining the rinses in a volumetric flask and making up to 200 mL with MeOH. The HP20 resin was extracted by sonication for 30 min with 3 × 50 mL MeOH. All extracts were filtered through a glass pipette, plugged with cotton wool, prior to analysis by LC-MS/MS.

#### 3.1.4. SPATTs

SPATT extracts, generated in studies carried out by Fux et al. [44,45], were analysed by LC-MS/MS, as described in Section 3.2. The SPATTs were deployed along the northwest of Ireland (Bruckless, Donegal, Ireland) at different depths (surface and 5 m) during an AZA toxic event. 

#### 3.1.5. Periodate Cleavage

Fifty microliters of 0.2 M sodium periodate solution was added to 100 µL of the HP20 resin extract from the 18 °C treatment and the reaction products analysed immediately by LC-MS/MS. 

#### 3.1.6. Impact of Growth Media

For the growth media experiment 10 mL of stock culture (in the exponential phase) was inoculated into 90 mL media, contained in 250 mL culture flasks (*n* = 3), and placed in incubators with a photoperiod of 12:12 L:D, a light intensity of 32 µmol m^−2^ s^−1^, and at 18 °C. Four media were tested; L1 [46] but with no silicates (Na_2_SiO_3_.9H_2_O), f/2 [47] with no silicates, f10k [49], and a diluted (2-fold) f10k.

To rule out matrix interferences in the LC-MS, matrix matched standards were prepared by adding 100 µL of each media to 50 µL of a CRM standard containing AZA1 and -2. The samples were vortex mixed for 0.5 min and stored at −18 °C until LC-MS/MS analysis.

#### 3.1.7. Impact of Photoperiod

For the photoperiod experiment 10 mL stock culture (in the exponential phase) was inoculated into 90 mL media (f10k) contained in 250 mL culture flasks (*n* = 3) and placed in incubators with photoperiods of 12:12, 16:8, and 8:16 L:D, a light intensity of 32 µmol m^−2^ s^−1^, and at 18 °C. 

### 3.2. Mass Spectrometry

Analysis was performed on an Acquity UPLC coupled to a Xevo G2-S QToF (Waters, Manchester, UK), operated in positive MS^e^ (200−1200 *m/z*) and MS/MS modes (AZA7 and isomer, *m/z* 858.5; AZA11 and isomer, *m/z* 872.5; AZA64, *m/z* 802.5; AZA64-P, *m/z* 882.5; AZA34-P, *m/z* 896.5; AZA35-P, *m/z* 910.5). Leucine encephalin was used as the reference compound. The cone voltage was 40 V, collision energy was 50 V, the cone and desolvation gas flows were set at 100 and 1000 L h^−1^, respectively, and the source temperature was 120 °C. Quantitation was performed in MS^e^ mode, using Targetlynx software. All toxins, other than AZA2 and -7 (plus isomer), were quantitated against AZA1.

Binary gradient elution was used, with phase A consisting of water and phase B of 95% acetonitrile in water (both containing 2 mM ammonium formate and 50 mM formic acid). The column used was a 50 mm × 2.1 mm i.d., 1.7 µm, Acquity UPLC BEH C18 (Waters, Wexford, Ireland). The gradient was from 30–90% B over 6 min at 0.3 mL min^−1^, held for 0.5 min, and returned to the initial conditions and held for 1 min to equilibrate the system. The injection volume was 2 µL and the column and sample temperatures were 25 °C and 6 °C, respectively.

### 3.3. Reagents

All solvents (pestican-grade) were from Labscan (Dublin, Ireland). Distilled water was further purified using a Barnstead nanopure diamond UV purification system (Thermo Scientific, Iowa, USA). Formic acid (≥98%), ammonium formate (>98%), Diaion HP-20 polymeric resin (≥0.25 mm), and sodium periodate were from Sigma-Aldrich (Steinheim, Germany). CRMs for AZA1 and -2 were obtained from the National Research Council (Halifax, NS, Canada) [26]. A noncertified calibrant standard for AZA7 was prepared, as described previously [50].

### 3.4. Statistical Analysis

Statistical calculations were carried out using a one-way ANOVA using Minitab^®^18 (Coventry, UK). Alpha was set at 0.05 (95% confidence) for all experiments. 

## 4. Conclusions

This study provides some useful information on maximizing toxin cell quotas and yields for the purposes of large scale culturing and isolation of AZAs, and it also provides valuable information on changes in toxin profiles over the various growth phases. AZA cell quotas are enhanced at a low temperature, confirming results from a previous study [34]. However, practical aspects for large scale cultivation at low temperatures have to be taken into account. The impact of different growth media has demonstrated that both higher cell densities and toxin cell quotas were achieved with a medium that has reduced amounts of various nutrients, and more targeted future experiments should address the physiological basis and explanations for this finding. Photoperiod also impacted, albeit in a relatively small way, cell growth and toxin quotas. The results from this study, and a previous study [34], indicate the influence of light (intensity and photoperiod) has relatively low impacts on toxin production. Overall, in this study, where single parameter experiments (temperature, growth media, and photoperiod) were performed, the highest AZA (AZA1+2) yields were achieved when cultures are grown at a lower temperature, using a f10k medium (with a reduced amount of nutrients), and a 12:12 L:D photoperiod. The results also show that highest yields are achieved in the later stages of the stationary phase.

The results, from this and previous studies [34,36,37], show there is huge variability in growth and toxin cell quota, even under presumptive identical environmental conditions. This clearly indicates there are other factors that are difficult, if not impossible, to control. Such a high and poorly understood variability of growth, cell yield, and toxin cell quota seriously hinders attempts to optimize a universal set of environmental conditions for maximum AZA batch culture yields. 

Identifying the polyketide genes responsible for AZA synthesis in *Azadinium* would be valuable to allow better control of toxin production via genetic manipulation, however, knowledge of the encoding genes responsible for toxin production in this species is currently lacking. 

This is the first study to assess the toxin profile of *A. spinosum* over the different growth phases (from lag to death). AZA1, -2, and -33 are produced during the exponential phase and the results indicate a metabolic shift in the biosynthetic pathway, once the stationary phase is reached, leading to the detection of AZA34, -35, and a newly identified AZA, named AZA64. Other AZAs were additionally detected; AZA7, -11, and their isomers, that are likely formed from the C3 hydroxylation of AZA1 and -2, respectively. Phosphorylated conjugates of AZA34, -35, and -64 were also found, and a reaction with sodium periodate strongly suggests the phosphorylation is occurring at the C20–C21 cis diol region of the molecule. The analysis of SPATT extracts, deployed during an AZA event, indicate that similar processes are occurring in the field. These results suggest the AZAs identified in this study, which are detected once the stationary phase is reached, should be considered in the analysis of shellfish and water samples during an AZA event.

In larger scale AZA production, loss of toxins due to adsorption (especially to plastic material), has to be taken into account. Moreover, although maximum yields are achieved in the late stationary phase, losses of AZA1 and -2 due to chemical conversions, in particular hydroxylation (to AZA7 and -11, respectively) should be considered.

The focus of this study was on AZAs; however, future work could focus on describing the full metabolome and identification of other novel compounds produced by *Azadinium*, which may have useful bioactive properties. Furthermore, the isolation and assessment of bioactivity of the new AZAs identified in this study could be attempted.

## Figures and Tables

**Table 1:** Fate of AZA1, -2, and -33 in the harvested (after ~4 months) 5 L culture flasks.

	18 °C			10 °C		
	AZA1	AZA2	AZA33	AZA1	AZA2	AZA33
% filtrate *	19.5	65.9	70.5	22.7	59.4	34.3
% adsorbed onto culture flask walls	6.2	24.7	29.5	14.7	35.7	65.7
% C3 hydroxylated ^#^	74.3	9.5		62.7	4.9	

* filter paper and HP20 resin extracts. ^#^ %C3 hydroxylated refers to hydroxylation of AZA1 and -2 to form AZA7 and -11 respectively.

**Table 2:** Exact Masses of [M + H]^+^ ions and calculated molecular formulae for AZA34-P, AZA35-P, AZA64, and AZA64-P in an *A. spinosum* HP-20 resin extract.

AZA	Molecular Formula ([M + H]^+^)	Measured *m*/*z* [M + H]^+^	Δ (ppm)
AZA34-P	C_45_H_71_NO_12_PO3	896.4547	−0.99
AZA35-P	C_46_H_73_NO_12_PO3	910.4738	2.21
AZA64	C_44_H_68_NO_12_	802.4742	0.74
AZA64-P	C_44_H_69_NO_12_PO3	882.4394	−0.61

**Table 3:** Concentrations of AZAs (µg L^−1^) detected in the harvested 5 L culture flasks after ~4 months.

							AZA						
Temperature	1	2	33	34	35	11	11 isomer	7	7 isomer	34-P	35-P	64	64-P
10 °C	5.8	6.4	0.6	1.4	0.6	0.2	0.3	4.8	4.9	3.6	1.2	0.6	1.0
18 °C	3.3	3.7	0.7	0.4	0.3	0.2	0.4	4.1	5.4	2.1	0.7	0.3	0.4

Note: All toxins, other than AZA2 and -7 (plus isomer), were quantitated against AZA1.

**Table 4:** Concentrations of AZAs (ng mL^−1^) in SPATTs deployed in Bruckless, Ireland in 2005 during an AZA event.

									AZA						
Depth (m)	1	2	33	34	35	11	11 isomer	7	7 isomer	34-P	35-P	64	64-P	1-P	2-P
0	1031.2	423.5	47.7	10.1	1.1	4.4	1.1	48.5	10.9	5.9	1.1	132.5	32.2	67.5	24.3
5	999.1	383.2	46.7	6.8	n.d.	2.8	0.6	1.1	10.1	3.1	0.5	86.6	34.9	58.5	38.5

n.d. = not detected. Note: All toxins, other than AZA2 and AZA7 (plus isomer), were quantitated against AZA1.

## Supplementary Materials

The following are available online at https://www.mdpi.com/1660-3397/17/9/489/s1, Figure S1: LC-MS analysis of L1, f/2, and f10k media spiked with a CRM, Figure S2: LC-MS chromatograms of AZA64 and AZA64-P prior to and after treatment with periodate, Figure S3: LC-MS chromatograms of AZA34 and AZA34-P prior to and after treatment with periodate, Figure S4: LC-MS chromatograms of AZA35 and AZA35-P prior to and after treatment with periodate, Figure S5: Figure 2 zoomed in for AZA34, -35, -64, and their phosphorylated conjugates, Figure S6: LC-MS chromatograms and spectra of AZA7 and isomer, Figure S7: LC-MS chromatograms and spectra of AZA11 and isomer, Figure S8: Figure 2 zoomed in for AZA7, -11, and their isomers, Figure S9: LC-MS chromatograms and spectra of AZA1 and AZA1-P, Figure S10: LC-MS chromatograms and spectra of AZA2 and AZA2-P, Table S1: Cell counts and quotas in the 18 °C culture flask, Table S2: Cell counts and quotas in the 10 °C culture flask, Table S3: Differences in nutrient compositions between L1, f/2, and f10k, Table S4: Relative (%) concentrations of AZAs to AZA1 in culture and SPATT extracts.
