# Supplementary material for: Effects of Temperature, Growth Media, and Photoperiod on Growth and Toxin Production of Azadinium spinosum
Source: Mar Drugs. 2019 Aug 22;17(9):489. doi: 10.3390/md17090489 (PMC6780083; doi:10.3390/md17090489)
Supplement: Supplementary file 1 [file marinedrugs-17-00489-s001.pdf]

Effects of temperature, growth media, and photoperiod on growth and toxin production of *Azadinium spinosum*

Jane Kilcoyne, Amy McCoy, Stephen Burrell, Bernd Krock and Urban Tillmann

Contents

Figure S1. LC-MS analysis of L1, f/2 and f10k media spiked with a CRM S2
Figure S2. LC-MS chromatograms of AZA64 and AZA64-P prior to and after treatment with periodate S3
Figure S3. LC-MS chromatograms of AZA34 and AZA34-P prior to and after treatment with periodate S4
Figure S4. LC-MS chromatograms of AZA35 and AZA35-P prior to and after treatment with periodate S5
Figure S5. Figure 2 zoomed in for AZA34, -35, -64 and their phosphorylated conjugates S6
Figure S6. LC-MS chromatograms and spectra of AZA7 and isomer S7
Figure S7. LC-MS chromatograms and spectra of AZA11 and isomer S8
Figure S8. Figure 2 zoomed in for AZA7, -11, and their isomers S9
Figure S9. LC-MS chromatograms and spectra of AZA1 and AZA1-P S10
Figure S10. LC-MS chromatograms and spectra of AZA2 and AZA2-P S11
Table S1. Cell counts and quotas in the 18 °C culture flask S12
Table S2. Cell counts and quotas in the 10 °C culture flask S13
Table S3. Differences in nutrient compositions between L1, f/2 and f10k S14
Table S4. Relative (%) concentrations of AZAs to AZA1 in culture and SPATT extracts S15

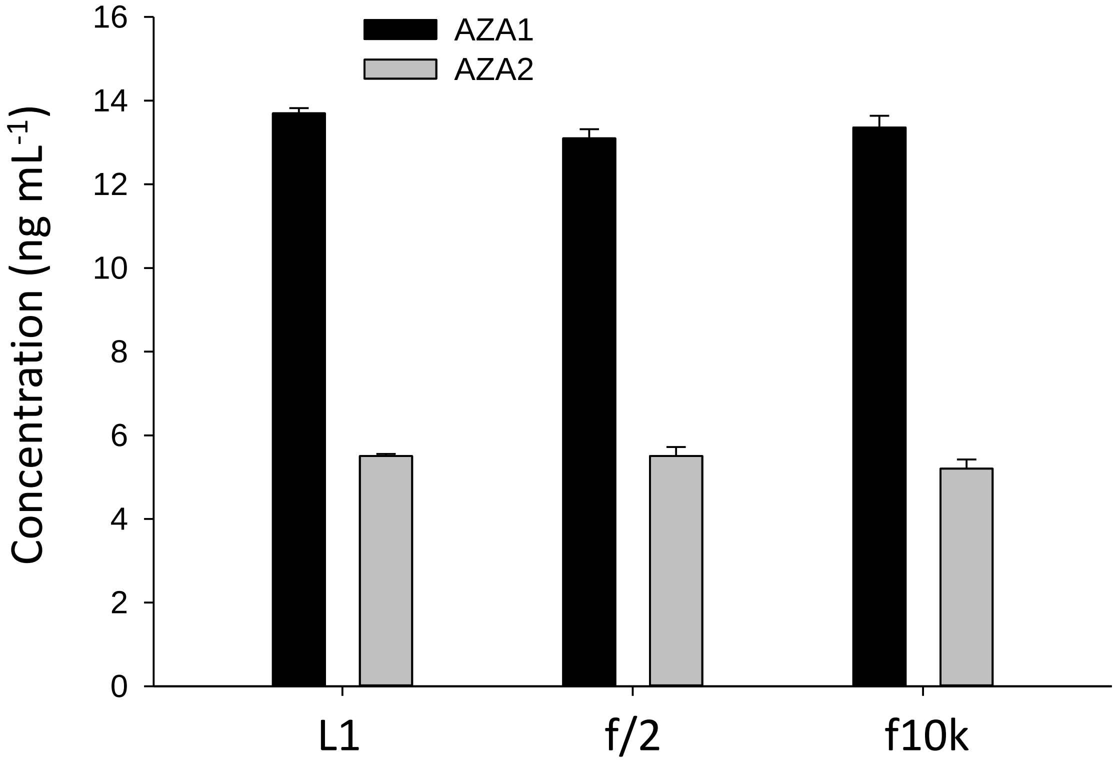

Figure S1. LC-MS analysis of L1, f/2 and f10k media spiked with a CRM containing AZA1 and -2.

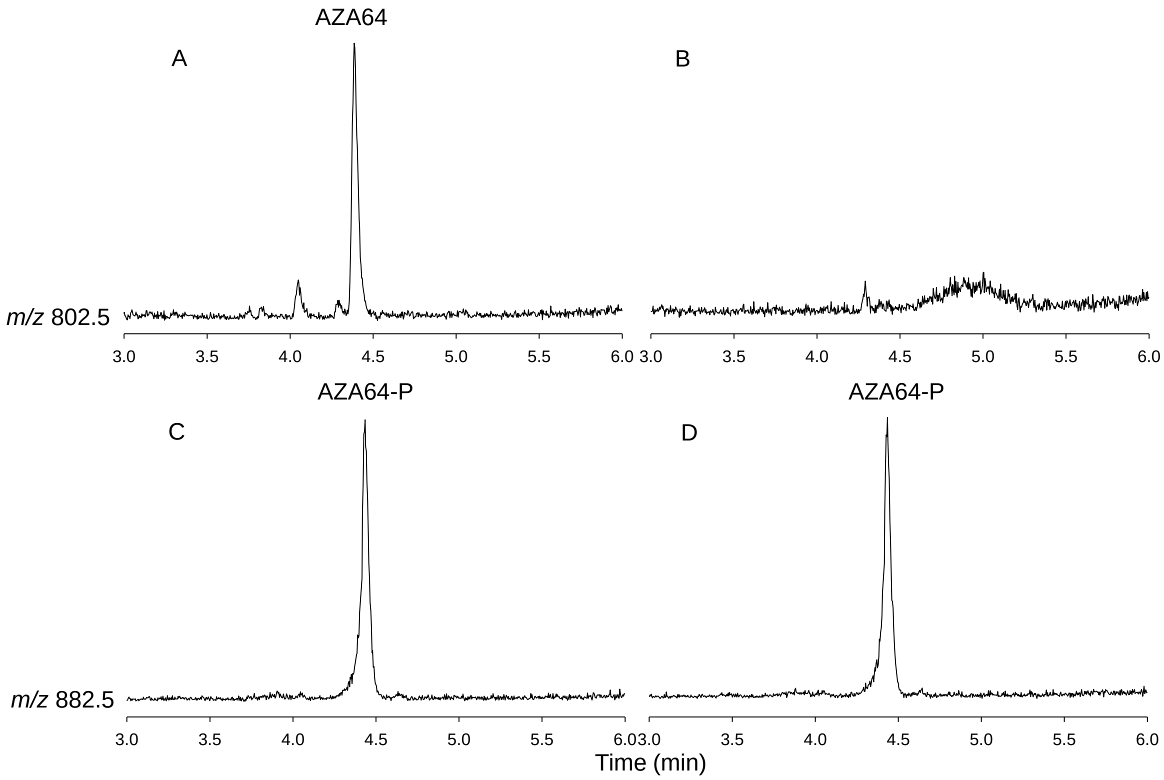

Figure S2. LC-MS analysis of *A. spinosum* HP20 resin extract showing chromatogram of A) AZA64, B) AZA64 after treatment with periodate, C) AZA64-P and D) AZA64-P after treatment with periodate.

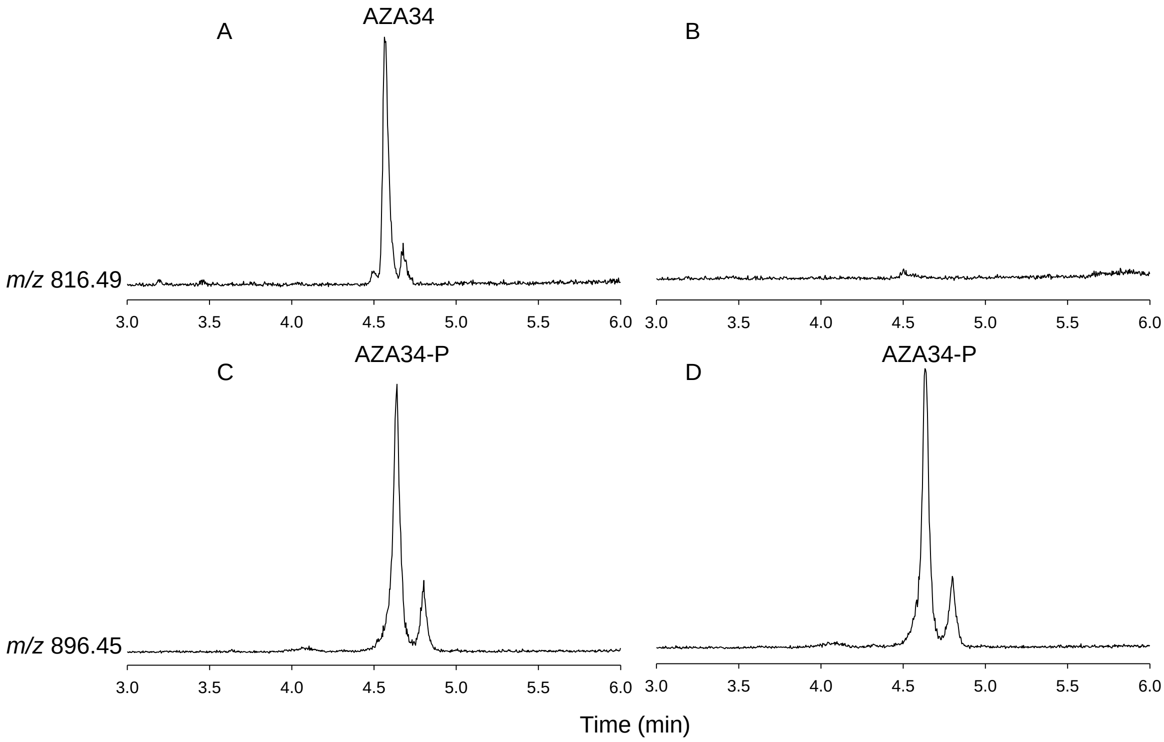

Figure S3. LC-MS analysis of *A. spinosum* HP20 resin extract showing chromatogram of A) AZA34, B) AZA34 after treatment with periodate, C) AZA34-P and D) AZA34-P after treatment with periodate.

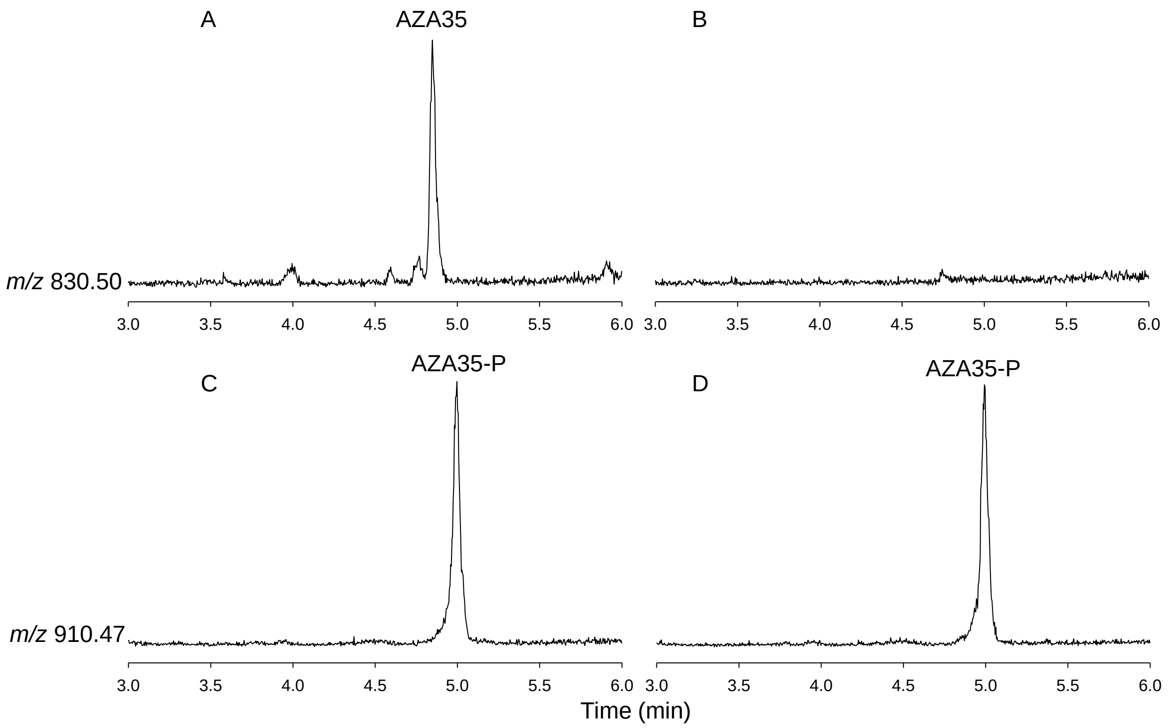

Figure S4. LC-MS analysis of *A. spinosum* HP20 resin extract showing chromatogram of A) AZA35, B) AZA35 after treatment with periodate, C) AZA35-P and D) AZA35-P after treatment with periodate.

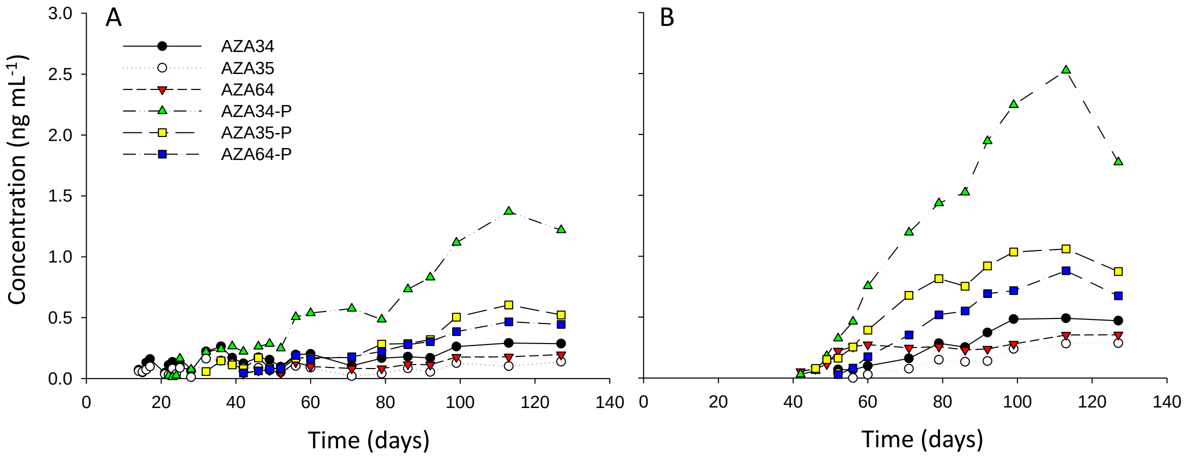

Figure S5. Figure 2 zoomed in - *A. spinosum* growth curves at A) 18 °C and B) 10 °C in the 5 L culture flasks showing concentration changes for AZA34, -35, -64 and their phosphorylated conjugates.

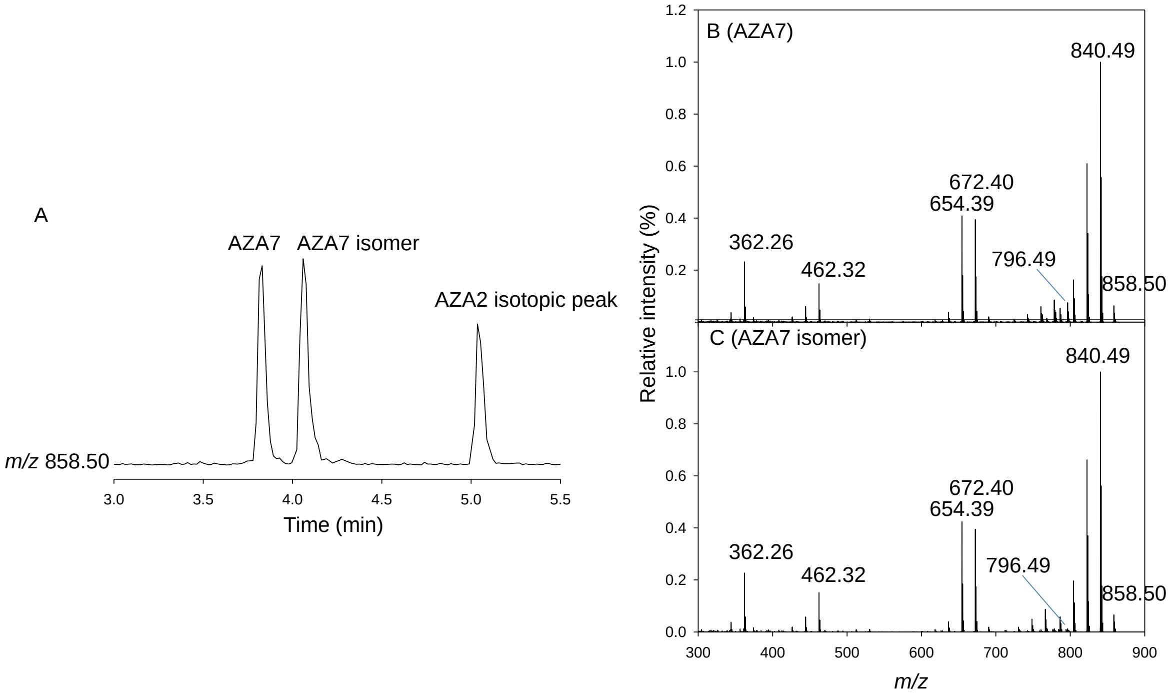

Figure S6. LC-MS analysis of *A. spinosum* HP20 extract showing A) chromatogram of AZA7 and isomer, B) AZA7 mass spectrum and C) AZA7 isomer mass spectrum (CE=50 V).

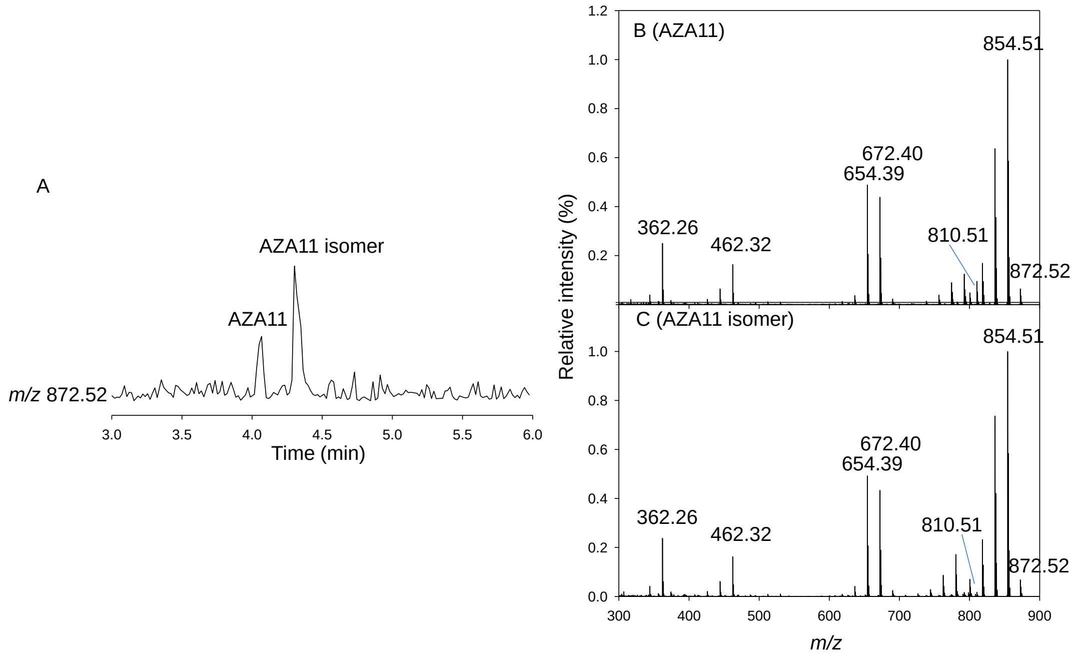

Figure S7. LC-MS analysis of *A. spinosum* HP20 extract showing A) chromatogram of AZA11 and isomer, B) AZA11 mass spectrum and C) AZA11 isomer mass spectrum (CE=50 V).

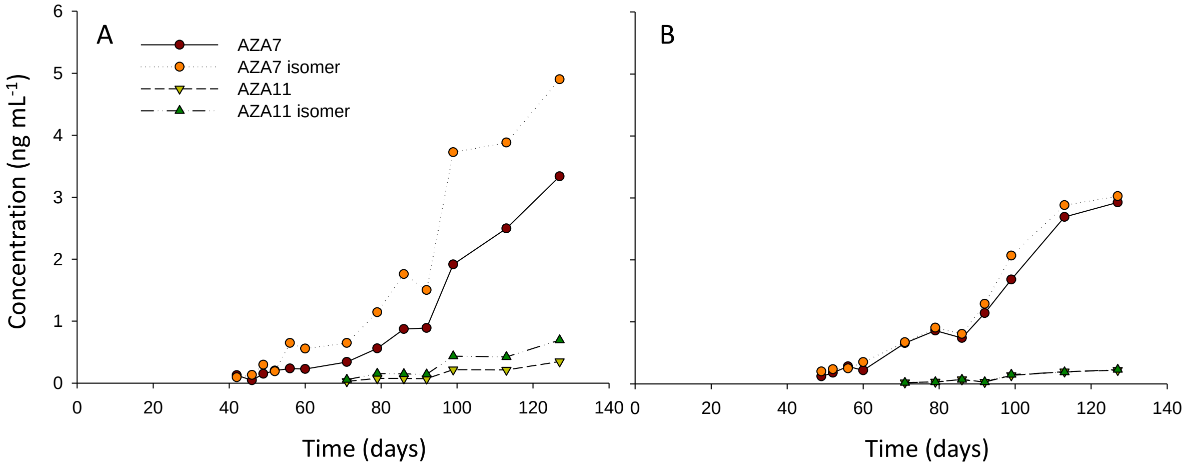

Figure S8. Figure 2 zoomed in - *A. spinosum* growth curves at A) 18 °C and B) 10 °C in the 5 L culture flasks showing concentration changes for AZA7, -11 and their isomers.

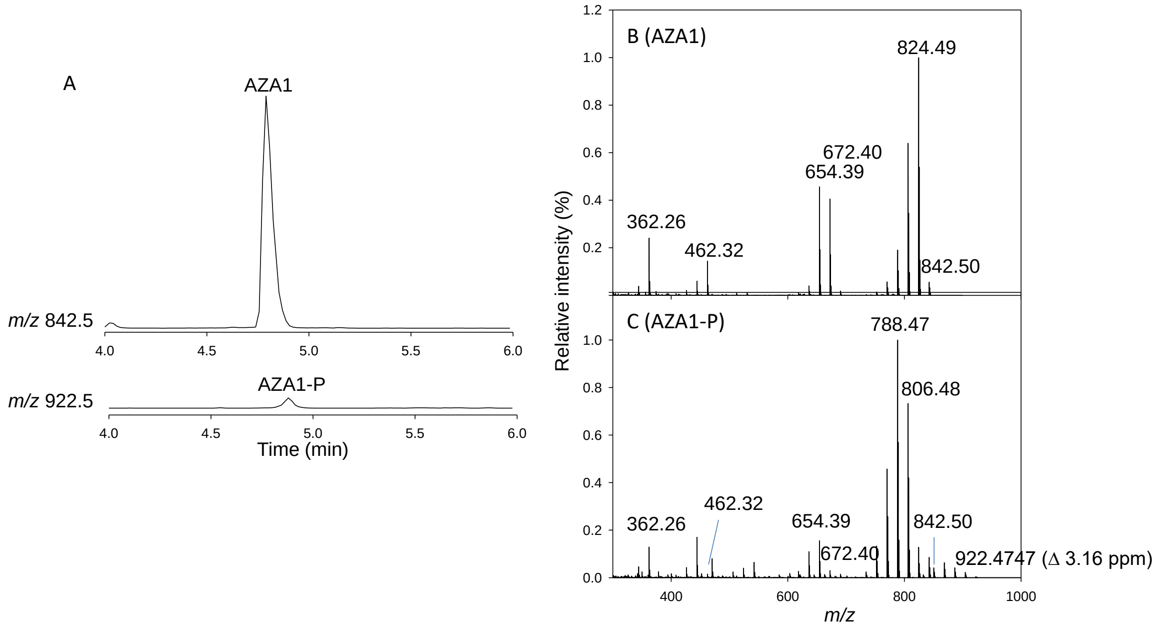

Figure S9. LC-MS analysis of a SPATT extract showing A) chromatogram of AZA1 and AZA1-P, B) AZA1 mass spectrum and C) AZA1-P mass spectrum (CE=50 V).

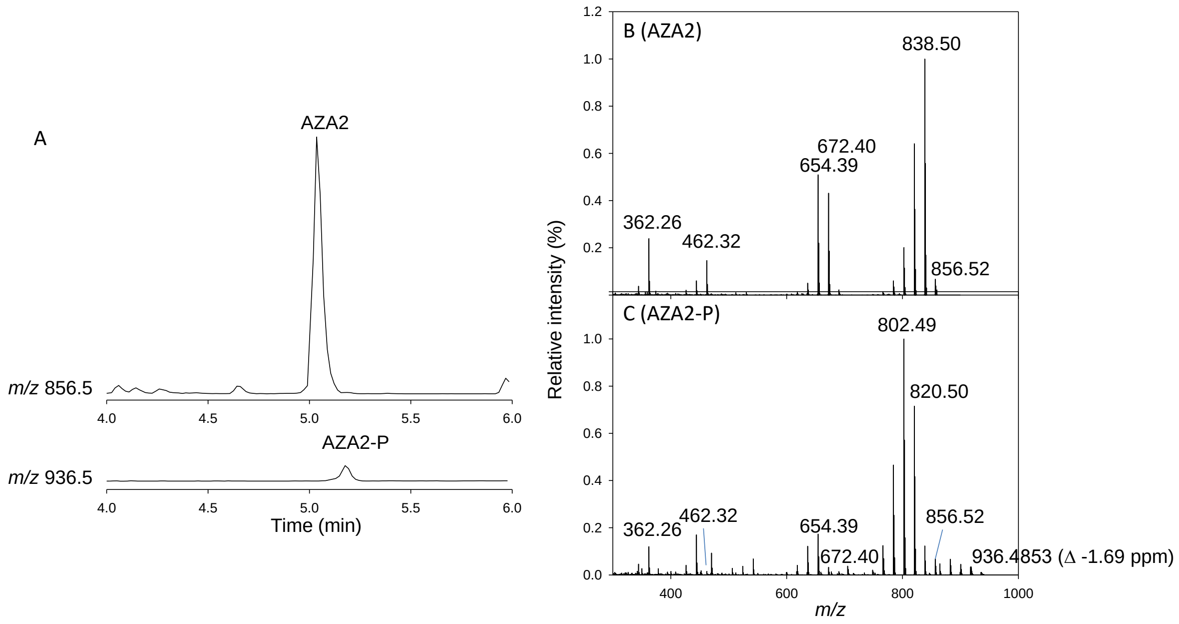

Figure S10. LC-MS analysis of a SPATT extract showing A) chromatogram of AZA2 and AZA2-P, B) AZA2 mass spectrum and C) AZA2-P mass spectrum (CE=50 V).

Table S1. Cell counts and quotas in the 18 °C culture flask

Time	Growth Phase	Cell counts (cells mL^{-1})	AZA1 (fg $cell^{-1}$)	AZA2 (fg $cell^{-1}$)	AZA33 (fg $cell^{-1}$)
0	E	14,464			
1	E	15,692			
2	E	24,412			
3	E	37,000			
4	E	53,250	8.0	7.2	4.5
			8.0	7.2	4.5
9	S	163,667	15.1	11.0	8.5
10	S	165,667	14.8	10.7	8.2
11	S	170,000	16.6	12.3	11.0
Average			15.5 ± 0.9	11.3 ± 0.9	9.2 ± 1.5
Fold increase from E phase			1.9	1.6	2.0
14	LS	173,333	50.0	27.9	11.4
15	LS	177,667	47.2	31.7	20.3
16	LS	170,668	41.8	26.2	19.3
17	LS	180,666	42.4	24.3	17.6
Average			43.8 ± 3.9	27.4 ± 3.1	19.1 ± 4.0
Fold increase from S phase			2.8	2.4	2.1

E = exponential, S = stationary, LS = late stationary

Table S2. Cell counts and quotas in the 10 °C culture flask

Time	Growth Phase	Cell counts (cells mL^{-1})	AZA1 (fg cell^{-1})	AZA2 (fg cell^{-1})	AZA33 (fg cell^{-1})
0	E	14,214			
1	E	14,138			
2	E	13,500			
3	E	12,719			
4	E	12,029			
9	E	19,095	54.4	40.6	19.4
10	E	16,520	41.6	21.4	14.6
11	E	19,091	30.6	21.8	17.2
14	E	26,438	68.9	49.3	19.7
15	E	26,733	47.5	34.8	23.2
16	E	29,857	39.7	37.6	24.0
17	E	32,769	60.0	43.0	30.0
21	E	47,222	55.8	47.5	20.2
22	E	50,250	54.9	49.7	22.3
23	E	62,857	77.6	61.5	26.3
24	E	73,167	82.0	58.5	25.8
25	E	76,000	74.9	59.1	29.1
28	E	102,000	91.9	76.5	37.4
			60.0 ± 18.2	46.3 ± 15.7	23.8 ± 6.1
32	S	174000	71.4	70.7	32.3
36	S	175000	99.1	102.5	51.0
Average			85.3	86.6	41.6
Fold increase from E phase			1.4	1.9	1.8
39	LS	166000	135.5	127.0	50.3
42	LS	175000	130.7	127.9	43.3
46	LS	167000	131.5	118.5	41.6
49	LS	170000	115.9	108.8	33.4
52	LS	164000	158.9	162.6	31.3
Average			134.5 ± 15.5	129.0 ± 20.3	40.0 ± 7.7
Fold increase from S phase			1.6	1.5	1.0

E = exponential, S = stationary, LS = late stationary

Table S3. Differences between L1, f/2 and f10k media.

	fold difference	
	L1 vs f10k	f/2 vs f10k
Nitrates	4.5	4.5
Phosphates	4.5	4.5
$FeCl_3.6H_2O$	4.5	4.5
$CuSO_4.5H_2O$	1.2	4.5
$ZnSO_4.7H_2O$	4.7	4.5
$CoCl_2.6H_2O$	5.3	4.5
$MnCl_2.2H_2O$	4.5	4.5
$Na_2MoO4.2H_2O$	14.3	4.5
H_2SeO_3	1.1	none in f/2
$Na_2EDTA.2H_2O$	1.6	1.6
$NiSO_4.6H_2O$	none in f10k	none in f/2
Na_3VO_4	none in f10k	none in f/2
K_2CrO_4	none in f10k	none in f/2
Thiamine	same	same
Biotin	same	same
B12	same	same

Red = lower amounts in f10k, Green = higher amounts in f10k

Table S4. Relative (%) concentrations of AZAs to AZA1 in the harvested culture (5 L flasks) and SPATT extracts.

	Culture 10 °C	Culture 18 °C	SPATT (0 m)	SPATT (5 m)
AZA2	110.3	112.1	41.1	38.4
AZA33	10.3	21.2	4.6	4.7
AZA34	24.1	12.1	1.0	0.7
AZA35	10.3	9.1	0.1	0.0
AZA11	3.4	6.1	0.4	0.3
AZA11 isomer	5.2	12.1	0.1	0.1
AZA7	82.8	124.2	4.7	0.1
AZA7 isomer	84.5	163.6	1.1	1.0
AZA34-P	62.1	63.6	0.6	0.3
AZA35-P	20.7	21.2	0.1	0.1
AZA64	10.3	9.1	12.8	8.7
AZA64-P	17.2	12.1	3.1	3.5
AZA1-P	n.d.	n.d.	6.5	5.9
AZA2-P	n.d.	n.d.	2.4	3.9

n.d. = not detected
